# Chloride alterations in hospitalized patients: Prevalence and outcome significance

**DOI:** 10.1371/journal.pone.0174430

**Published:** 2017-03-22

**Authors:** Charat Thongprayoon, Wisit Cheungpasitporn, Zhen Cheng, Qi Qian

**Affiliations:** 1 Division of Nephrology and Hypertension, Department of Medicine, Mayo Clinic, Rochester, Minnesota, United States of America; 2 Department of Anesthesiology, Mayo Clinic Rochester, Minnesota, United States of America; 3 National Clinical Research Center of Kidney Disease, Jinling Hospital, Nanjing University School of Medicine, Nanjing, China; National Yang-Ming University, TAIWAN

## Abstract

Serum Cl (sCl) alterations in hospitalized patients have not been comprehensively studied in recent years. The aim of this study is to investigate the prevalence and outcome significance of (1) sCl alterations on hospital admission, and (2) sCl evolution within the first 48 hr of hospital admission. We conducted a retrospective study of all hospital admissions in the years 2011–2013 at Mayo Clinic Rochester, a 2000-bed tertiary medical center. Outcome measures included hospital mortality, length of hospital stay and discharge disposition. 76,719 unique admissions (≥18 years old) were studied. Based on hospital mortality, sCl in the range of 105–108 mmol/L was found to be optimal. sCl <100 (n = 13,611) and >108 (n = 11,395) mmol/L independently predicted a higher risk of hospital mortality, longer hospital stay and being discharged to a care facility. 13,089 patients (17.1%) had serum anion gap >12 mmol/L; their hospital mortality, when compared to 63,630 patients (82.9%) with anion gap ≤12 mmol/L, was worse. Notably, patients with elevated anion gap displayed a progressively worsening mortality with rising sCl. sCl elevation within 48 hr of admission was associated with a higher proportion of 0.9% saline administration and was an independent predictor for hospital mortality. Moreover, the magnitude of sCl rise was inversely correlated to the days of patient survival. In conclusion, serum Cl alterations on admission predict poor clinical outcomes. Post-admission sCl increase, due to Cl-rich fluid infusion, independently predicts hospital mortality. These results raise a critical question of whether iatrogenic cause of hyperchloremia should be avoided, a question to be addressed by future prospective studies.

## Introduction

Chloride (Cl) is the most abundant anion in extracellular fluid, playing a fundamental role in the maintenance of osmotic pressure, water distribution and acid–base balance [1]. Cl channels are expressed in almost all cells in the body. Dysfunctions in the Cl channel result in a broad spectrum of diseases [2].

Variations in electrolyte concentration drive changes in the ionization state of water molecules that alter the hydrogen ion concentration [H^+^] and pH. Three independent factors (strong ion difference [SID], A_TOT_ (total weak non-volatile acids) and pCO_2_) determine the plasma pH by changing the degree of water dissociation. An excess of plasma anions relative to cations gives rise to acidosis, as disproportionate anion accumulation causes a fall in the [OH^-^]. By contrast, an excess of cations lowers the [H^+^], leading to alkalosis [3]. Of the three independent pH determinants, SID is dominant. SID is, however, cumbersome to calculate and some SID components are not routinely measured in practice. Recently, serum sodium (Na)-Cl difference (Diff_Na-Cl_) and Cl:Na ratio have been introduced as surrogates for SID. They exhibit adequate receiver-operating characteristic curves in determining acid-base status [4, 5].

Hyperchloremia is a common feature in sepsis and a frequent etiology of metabolic acidosis in critically ill patients [6]. Often, the source of the acidosis is multiple and at least partly iatrogenic because 0.9% saline resuscitation is routinely used for sepsis and shock patients. Saline infusion can produce hyperchloremia and metabolic acidosis. Mobilization of endogenous Cl from tissue/cell may also contribute to hyperchloremia [7]. Additionally, acidosis in critically ill patients can be due to the presence of anions that are not routinely measured (unmeasured anions, UMAs), including lactate, β-hydroxybutyrate, acetoacetate, anions associated with uremia and other toxins. UMAs can be quantified by calculating the anion gap (AG) [8] or strong ion gap (SIG) [9, 10]. AG corrected for albumin is an excellent surrogate for SIG [5].

Studies on the clinical impact of serum Cl (sCl) alterations are limited. Several publications show that hypo- and hyperchloremia can both be associated with increased hospital mortality in critically ill patients [11–15], patients with severe sepsis and septic shock [15, 16], and in post-surgery patients [14, 17, 18]. The studies, however, are limited by lack of data on anion-gap and serum Na alterations. A recent study by Young et al. [19] compared buffered versus non-buffered fluid administration for intensive care units (ICUs) patients and found no difference in the AKI occurrence and hospital mortality. The study, however, was commenced in the ICUs after the participants were managed in various care settings (including the operation rooms, regular hospital units and ICUs of another hospital) where they likely had received fluids (amount unknown). There was no data on the patient volume status and no data on hyperchloremia with the study fluid (average of 2L) administration. Thus, it is unclear whether the study results are related to serum Cl alterations. There has not been a comprehensive study on the effects of sCl alterations upon hospital admission and consequences of sCl evolution after admission.

The aim of this study is to investigate the prevalence and outcome significance of (1) sCl alterations upon hospital admission, and (2) sCl evolution within 48 hr following the hospital admission in recent years.

## Materials and methods

**Fig 1:**
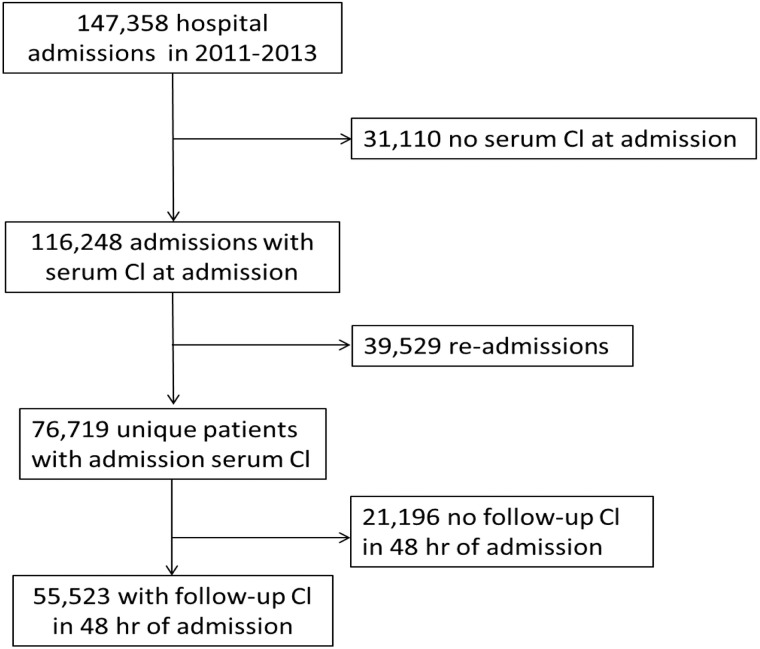
The study flow chart.

**Fig 2:**
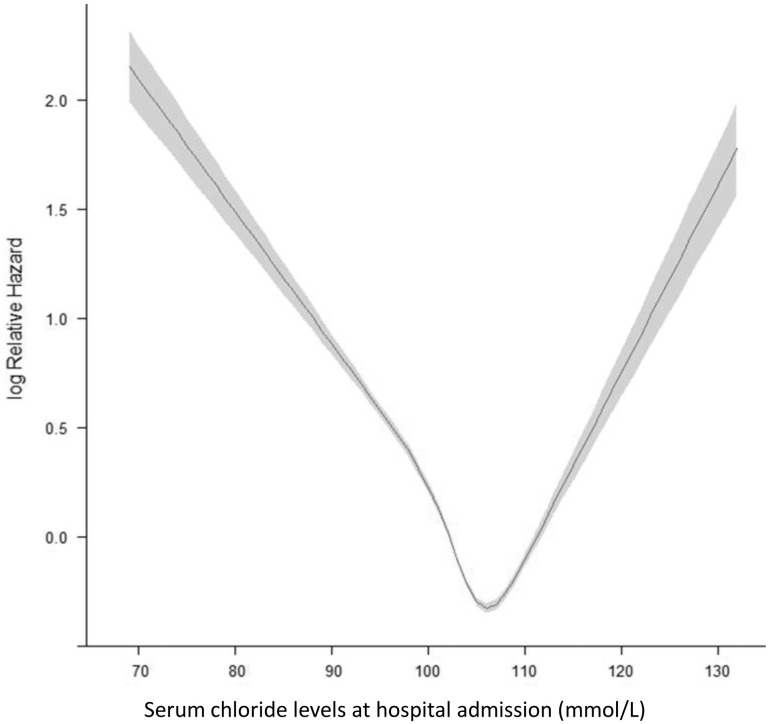
Hospital mortality analyzed by restricted cubic spline.

The Institutional Review Board approved the study and waver of consent. All participants had provided Mayo Clinic with research authorization. Participant records were de-identified and analyzed anonymously. Adults (age >18 years) admitted to Mayo Clinic Rochester between January 1, 2011 and December 31, 2013 were enrolled (Fig 1). Patients without admission sCl (≤24hr of admission) were excluded. For patients with multiple admissions, data from the first admission were analyzed. Charlson comorbidity index [20] was computed for each patient at the index admission. Clinical data, including principal diagnosis based on the ICD-9 (International Classification of Diseases, 9th Revision) codes, were extracted from our institutional electronic database. sCl values were grouped, based on hospital mortality data (Fig 2) into: <95, 95–100, 100–105, 105–108 (optimal), 108–113, 113–118 and >118 mmol/L. Acid-base status was determined (Box 1). Diff_Na-Cl_ and Cl:Na ratio were used as surrogates for SID [4, 5], and AG (Na-Cl-HCO_3_) used to estimate UMAs.

**Box 1. Definition of acid-base alterations:[5, 42, 43]**

- Hypochloremic alkalosis:
  Cl:Na ratio <0.75
  Diff_Na–Cl_ >37 mmol/L
- Hyperchloremic acidosis:
  Cl:Na ratio >0.77
  Diff_Na–Cl_: <32 mmol/L
- Mixed alterations:
  AG >12 mmol/L
  Cl:Na ratio 0.75–0.77
  Diff_Na–Cl_ 32–37 mmol/L

### Serum Cl measurement

In out institution, sCl is measured using the Roche Cobas 8000 ISE analytics, which utilizes an indirect potentiometric assay with an ion-selective electrode (ISE). The electrode has a selective membrane in contact with both the test solution (patient’s sample) and an internal filling solution (containing the test ion at a fixed concentration). The membrane electromotive force is determined by the difference in concentration of the test ion in the two solutions. In our experience, such method for sCl measurement has imprecision (coefficient of variation) of 1–2% across the measurable range. The quality control program in our clinical laboratory is designed to minimize between instrument bias and between lot shifts to ≤3 mmol/L.

### Statistical analysis

Continuous and categorical variables are reported as means±SDs and counts with percentages, respectively. Baseline characteristics among groups were compared, using ANOVA for continuous and Chi-square for categorical variables. Missing data were not imputed; lower counts were reported. Hospital mortality and sCl were modeled using smoothing splines to allow for non-linear effects. The restricted cubic spline with 4 knots was used with sCl when fitting the model; plot constructed using the design library, R version 3.0 (Free software Foundation, California) [21]. Multivariable logistic and linear regressions were performed to assess the associations between sCl and clinical outcomes (mortality, length of hospital stay [LOS] and discharge disposition). Odds ratio (OR) and 95% confidence interval (CI) were reported. Two-tailed p of <0.05 was considered significant. Unless specified, JMP statistical software (version 9.0, SAS Institute Inc., NC) was used for analyses.

We hypothesized that patients with sCl increase (>4 mmol/L) received higher proportion of Cl-rich fluids (0.9% saline) than patients with minimal sCl change (-2 to 0 mmol/L). However, comparison of the two groups using the whole dataset would likely cause over-power, resulting a statistical significant but (possibly) clinically meaningless or misleading result. To avoid such a possibility, we did a preliminary testing of 15 patients in each of the two groups (samples randomized) to assess the difference in the percentage of 0.9% saline between the two groups. The testing showed a difference to be approximately 20%, and a power calculation yielded a total of 150 patients needed to generate a >85% power. We, therefore, included 200 patients (100 patients in each group of randomized sample) for analysis.

## Results

### 1. Patients characteristics

**Fig 3:**
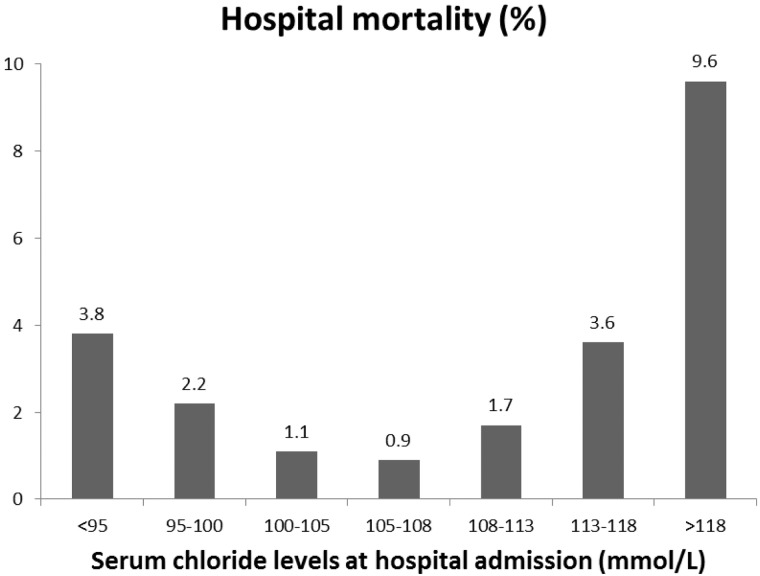
Hospital mortality in percentage (%) among patients with various admission Cl levels.

76,719 unique patients from a total of 147,358 hospital admissions were enrolled. 55,523 (72.4%) had repeat sCl measurements within 48 hours (Fig 1). 23.6% (n = 18,066) of the 76,719 had an optimal sCl level (105–108 mmol/L) based on hospital mortality (Table 1, Figs 2 and 3). 61.6% (n = 47,258) patients had sCl <105 mmol/L, and 14.9% (n = 11,395) patients had sCl >108 mmol/L on admission.

**Table 1:** Baseline clinical characteristics.

Variables	All	Admission serum chloride level (mmol/L)							
		<95	95–100	100–105	105–108	108–113	113–118	>118	p
N	76,719	3,360	10,251	33,647	18,066	9,656	1,490	249	
Age (year)	61.1±17.8	66±16	64±17	60±18	60±18	61±18	61±17	62±19	<0.001
Male	40,515 (53)	1,602 (48)	5,498 (54)	18,519 (55)	9,190 (51)	4,864 (50)	717 (48)	125 (50)	<0.001
Caucasian	71,229 (93)	3,136 (93)	9,483 (93)	31,319 (93)	16,762 (93)	8,956 (93)	1,348 (90)	225 (90)	
Principal Diagnosis Cardiovascular Endocrine/Metabolic Gastrointestinal Renal Disease Hematology/Oncology Infectious Disease Respiratory Injury/poisoning Other	16,275 (21) 2,084 (3) 7,118 (9) 2850 (4) 11,712 (15) 2,399 (3) 3,085 (4) 11,823 (15) 19,373 (25)	618 366 415 162 334 220 340 351 554	1,870 432 1,148 405 1,430 546 820 1,421 2,179	6,138 800 3,086 1,273 5,796 893 1,266 5,067 9,328	3,898 291 1,495 607 2,919 361 416 3,051 5,028	3,116 154 821 337 1,082 264 213 1,623 2,046	588 30 126 49 138 80 22 238 219	47 11 27 17 13 35 8 72 19	<0.001
Charlson Score	1.8±2.3	2.5±2.7	2.3±2.6	1.7±2.3	1.6±2.2	1.6±2.2	1.6±2.2	1.8±2.3	<0.001
eGFR (ml/min/1.73m^2^)	78.3±28.4	69±33	74±31	80±27	80±27	76±29	74±32	69±39	<0.001
Na (mmol/L)	138.0±4.0	129.1±5.9	134.8±3.4	137.9±2.7	139.6±2.5	140.6±2.7	142.3±3.5	145.6±7.8	<0.001
K (mmol/L)	4.2±0.6	4.2±0.8	4.2±0.6	4.3±0.5	4.2±0.5	4.2±0.6	4.1±0.7	3.8±1.1	<0.001
HCO_3_ (mmol/L)	25.2±3.6	27.0±5.5	26.4±3.9	25.7±3.0	24.7±2.9	23.0±3.2	21.0±3.6	19.0±5.8	<0.001
Diff_Na-Cl_ (mmol/L)	34.8±3.7	38.6±5.1	37.3±3.3	35.6±2.6	33.7±2.5	31.3±2.8	28.1±3.5	24.1±6.7	<0.001
Cl: Na ratio	0.75±0.03	0.70±0.03	0.72±0.02	0.74±0.01	0.76±0.01	0.78±0.02	0.80±0.02	0.84±0.04	<0.001
Anion Gap (mmol/L)	9.6±3.4	11.6±4.9	10.8±3.6	9.9±3.1	9.0±3.0	8.3±3.3	7.0±4.1	5.1±6.9	<0.001
Albumin (g/dL), n = 10,576	3.5±0.7	3.4±0.7	3.5±0.7	3.6±0.7	3.6±0.7	3.4±0.7	3.1±0.7	3.0±0.8	<0.001

Continuous data are presented as mean ± SD; categorical data are presented as count (%)

Acid-base profile was assessed. With increasing sCl, Diff_Na-Cl_ declined progressively from 38.6±5.1 to 24.1±6.7 mmol/L; Cl:Na ratios increased from 0.70±0.03 to 0.84±0.04. AG and HCO_3_ declined (Table 1). In patients with sCl <95 mmol/L, their Diff_Na-Cl_ was 38.6±5.1, Cl:Na ratio 0.70±0.03, HCO_3_ 27.0±5.5 and AG 11.6±4.9 mmol/L, suggesting the presence of mixed hypochloremic alkalosis and AG acidosis. In those with sCl >118 mmol/L, Diff_Na-Cl_ was 24.1±6.7, Cl:Na ratio 0.84±0.04, HCO_3_ 19.0±5.8 and AG 5.1±6.9 mmol/L. These changes are consistent with a dominant presence of hyperchloremic acidosis. Acid-base alterations were further delineated based on the patients’ AG (detailed below).

### 2. Clinical outcomes

#### 2-a. Hospital mortality

Initial determination of hospital mortality (Fig 2) showed that the lowest mortality risk was with sCl in the range of 105–108 mmol/L (designated as optimal sCl value). Concordant changes in the hospital mortality (in %) were noted (Fig 3).

Logistic regression models were built to determine the contribution of sCl alterations to the risk of mortality (Table 2). In both the unadjusted and adjusted models (Models 1 and 2), the ORs for hospital mortality were significantly elevated in sCl <100 and >108 mmol/L. Dysnatremia, when disproportionate to Cl alterations, can independently alter patient mortality [22, 23]. We therefore further adjusted the model 2 for Na indifferences (model 3). The ORs remained significant. These results are consistent with sCl alterations (outside 100–108 mmol/L) being an independent predictor for hospital mortality.

**Table 2:** Clinical outcomes.

Outcome	Admission serum chloride level (mmol/L)						
	<95	95–100	100–105	105–108	108–113	113–118	>118
Hospital mortality	127 (3.8)	226 (2.2)	355 (1.1)	164 (0.9)	164 (1.7)	53 (3.6)	24 (9.6)
Mortality, OR (95%CI)							
Model 1: unadjusted	4.29 (3.39–5.42)	2.46 (2.01–3.02)	1.16 (0.97–1.40)	1 (ref)	1.89 (1.52–2.35)	4.03 (2.91–5.47)	11.64 (7.27–17.89)
Model 2#	2.63 (2.06–3.34)	1.66 (1.35–2.05)	1.11 (0.92–1.35)	1 (ref)	1.61 (1.29–2.01)	2.96 (2.12–4.05)	6.34 (3.84–10.09)
Model 3: Model 2 and Na	2.14 (1.59–2.87)	1.51 (1.21–1.89)	1.08 (0.89–1.30)	1 (ref)	1.65 (1.32–2.06)	3.14 (2.25–4.32)	7.26 (4.35–11.67)
Hospital LOS	5 (3–9)	5 (3–8)	4 (3–6)	4 (3–7)	5 (3–7)	6 (5–9)	6 (4–12)
LOS, relative prolongation (95% CI)							
Model 1: unadjusted	1.28 (1.25–1.32)	1.13 (1.11–1.15)	0.98 (0.97–0.99)	1 (ref)	1.15 (1.13–1.17)	1.45 (1.40–1.50)	1.53 (1.40–1.67)
Model 2#	1.25 (1.22–1.29)	1.11 (1.09–1.12)	0.98 (0.97–1.002)	1 (ref)	1.13 (1.11–1.15)	1.39 (1.34–1.45)	1.45 (1.33–1.58)
Model 3: Model 2 and Na	1.29 (1.26–1.34)	1.12 (1.10–1.14)	0.99 (0.97–1.01)	1 (ref)	1.12 (1.10–1.14)	1.39 (1.33–1.43)	1.42 (1.31–1.55)
Hospital survivor (n = 75,606)	3,233	10,025	33,292	17,902	9,492	1,437	225
Discharge disposition							
- Home	2,203 (68)	7,398 (74)	27,123 (81)	14,590 (81)	7,558 (80)	1,072 (75)	139 (62)
- Home Health Care	305 (9)	856 (9)	1,897 (6)	994 (6)	568 (6)	96 (7)	12 (5)
- In-hospital rehab^+^	13 (0.4)	45 (0.4)	91 (0.3)	75 (0.4)	36 (0.4)	12 (0.9)	1 (0.4)
- Skilled Nursing facility ^+^	686 (21)	1,680 (17)	4,066 (12)	2,191 (12)	1,299 (14)	247 (17)	69 (31)
- Swing bed^+^	26 (0.8)	46 (0.4)	115 (0.3)	52 (0.3)	31 (0.3)	10 (0.7)	4 (2)
Discharge to short or long term care facility, OR (95% CI)							
Model 1: unadjusted	1.94 (1.77–2.13)	1.44 (1.35–1.54)	0.99 (0.94–1.04)	1 (ref)	1.13 (1.05–1.21)	1.55 (1.34–1.78)	3.29 (2.47–4.35)
Model 2#	1.46 (1.31–1.62)	1.16 (1.08–1.26)	0.97 (0.92–1.03)	1 (ref)	1.16 (1.08–1.26)	1.89 (1.61–2.20)	3.53 (2.53–4.88)
Model 3: Model 2 and Na	1.48 (1.30–1.69)	1.18 (1.08–1.28)	0.98 (0.92–1.04)	1 (ref)	1.16 (1.07–1.26)	1.88 (1.60–2.19)	3.49 (2.50–4.85)

^#^model 2: Adjusted for age, sex, Charlson comorbidities score, eGFR and principal diagnosis.

^+^discharge to short or long term care facility

#### 2-b. Length of Hospital Stay (LOS) and discharge disposition

LOS and discharged to a care facility are associated with patients’ long-term morbidity and mortality[24]. In fully adjusted models, sCl <100 and >108 mmol/L were independently associated with elevated risks for both (Table 2). It should be noted that LOS and hospital discharge disposition can be affected by multiple factors and may not be strongly influenced by serum Cl alterations.

### 3. Patients with Anion Gap ≤ or >12 mmol/L

As the data from the entire cohort suggested the presence of mixed acid-base alterations, we grouped patients based on their serum AG. The AG values were not adjusted for serum albumin because only 13.8% of the study patients had admission albumin values and the albumin variations were small (range: 3.0–3.6 g/dL) and could not have altered AG to a large degree [25].

#### 3-a. Characteristics of patients with AG ≤12 mmol/L

82.9% (n = 63,630) of the patients had AG ≤12 mmol/L. Patients with lower sCl were older and had higher Charlson scores (Table 3). With increasing sCl, the Diff_Na-Cl_ declined from 37.5±4.8 to 23.2±6.1 mmol/L, Cl:Na ratio rose from 0.71±0.03 to 0.84±0.04, and HCO_3_ declined from 28.5±5.1 to 19.5±5.5 mmol/L. These results are consistent with the presence of alkalosis in patients with hypochloremia and acidosis with hyperchloremia.

**Table 3:** Baseline clinical characteristics of patients with AG ≤ 12.

Variables	All	Admission serum chloride level (mmol/L)							
		<95	95–100	100–105	105–108	108–113	113–118	>118	p
N	63,630	2,178	7,368	27,742	15,985	8,755	1,380	222	
Age (year)	61.4±17.7	69±15	65±17	61±18	60±18	61±18	61±17	62±19	<0.001
Male	33,483 (53)	998 (46)	3,922 (53)	15,291 (55)	8,113 (51)	4,384 (50)	662 (48)	113 (51)	<0.001
Caucasian	59,199 (93)	2,044 (94)	6,859 (93)	25,862 (93)	14,852 (93)	8,127 (93)	1,254 (91)	201 (91)	0.01
Principal Diagnosis Cardiovascular Endocrine/Metabolic Gastrointestinal Renal Disease Hematology/Oncology Infectious Disease Respiratory Injury/poisoning Other	13,113 (21) 1,500 (2) 5,844 (9) 2,166 (3) 9,729 (15) 1,872 (3) 2,617 (4) 10,060 (16) 16,729 (26)	403 218 253 77 227 138 277 221 364	1,275 246 836 242 1,040 392 670 1,076 1,591	4,696 631 2,553 1,011 4,785 719 1,076 4,256 8,015	3,309 250 1,316 509 2,566 304 371 2,743 4,617	2,821 126 745 273 974 226 196 1,478 1,916	563 21 116 41 127 65 21 218 208	46 8 25 13 10 28 6 68 18	<0.001
Charlson Score	1.8±2.3	2.5±2.7	2.3±2.7	1.7±2.3	1.6±2.2	1.6±2.2	1.6±2.2	1.7±2.2	<0.001
eGFR (ml/min/1.73m^2^)	79.8±26.8	74±29	76±29	82±26	81±26	78±28	76±30	73±38	<0.001
Na (mmol/L)	137.8±3.8	128.1±5.8	134.3±3.3	137.6±2.6	139.3±2.4	140.4±2.5	142.0±3.2	144.7±7.1	<0.001
K (mmol/L)	4.2±0.5	4.2±0.7	4.2±0.6	4.3±0.5	4.2±0.5	4.2±0.5	4.1±0.7	3.8±1.1	<0.001
HCO_3_ (mmol/L)	25.7±3.2	28.5±5.1	27.5±3.3	26.4±2.6	25.2±2.6	23.4±2.8	21.4±3.2	19.5±5.5	<0.001
Diff_Na-Cl_ (mmol/L)	34.3±3.4	37.5±4.8	36.7±3.1	35.3±2.5	33.5±2.4	31.0±2.7	27.8±3.2	23.2±6.1	<0.001
Cl: Na ratio	0.75±0.02	0.71±0.03	0.73±0.02	0.74±0.01	0.76±0.01	0.78±0.02	0.80±0.02	0.84±0.04	<0.001
Anion Gap (mmol/L)	8.5±2.4	8.9±2.5	9.2±2.2	8.9±2.2	8.3±2.3	7.6±2.6	6.4±3.4	3.7±5.9	<0.001
Albumin (g/dL), n = 7,866	3.5±0.7	3.3±0.6	3.4±0.7	3.6±0.7	3.6±0.7	3.4±0.7	3.1±0.7	2.9±0.8	<0.001

Continuous data are presented as mean±SD; categorical data are presented as count (%)

#### 3-b. Characteristics of patients with AG >12 mmol/L

17.1% (n = 13,089) of the patients had AG >12 mmol/L, consistent with the presence of UMAs (Table 4). For those with sCl <95 mmol/L, Diff_Na-Cl_ was 40.8±4.9 mmol/L, Cl:Na ratio 0.69±0.03, HCO_3_ 24.3±5.2 mmol/L and AG 16.5±4.3 mmol/L, consistent with the co-existence of UMA acidosis and hypochloremic alkalosis. For patients with high sCl (from 108 to >118 mmol/L), Diff_Na-Cl_ declined from 33.6±3.2 to 31.3±7.1 mmol/L, Cl:Na ratios rose from 0.77±0.02 to 0.80±0.04, and HCO_3_ reduced from 18.9±3.7 to 15.0±6.4 mmol/L, consistent with the co-existence of duo acidoses due to UMA and hyperchloremia.

**Table 4:** Baseline clinical characteristics of patients with AG > 12.

Variables	All	Admission serum chloride level (mmol/L)							
		<95	95–100	100–105	105–108	108–113	113–118	>118	p
N	13,089	1,182	2,883	5,905	2,081	901	110	27	
Age (year)	59.8±18.2	62±17	61±18	59±18	59±19	61±19	61±19	67±20	<0.001
Male	7,032 (54)	604 (51)	1,576 (55)	3,228 (55)	1,077 (52)	480 (53)	55 (50)	12 (44)	0.08
Caucasian	12,030 (92)	1,092 (92)	2,624 (91)	5,457 (92)	1,910 (92)	829 (92)	94 (85)	24 (89)	
Principal Diagnosis Cardiovascular Endocrine/Metabolic Gastrointestinal Renal Disease Hematology/Oncology Infectious Disease Respiratory Injury/poisoning Other	3,162 (24) 584 (4) 1,274 (10) 684 (5) 1,983 (15) 527 (4) 468 (4) 1,763 (13) 2,644 (20)	215 148 162 85 107 82 63 130 190	595 186 312 163 390 154 150 345 588	1,442 169 533 262 1,011 174 190 811 1,313	589 41 179 98 353 57 45 308 411	295 28 76 64 108 38 17 145 130	25 9 10 8 11 15 1 20 11	1 3 2 4 3 7 2 4 1	<0.001
Charlson Score	1.9±2.4	2.5±2.8	2.2±2.6	1.7±2.2	1.8±2.3	2.0±2.4	1.9±2.5	2.1±2.4	<0.001
eGFR (ml/min/1.73m^2^)	70.7±34.2	58±37	69±36	75±32	73±32	62±36	51±37	37±30	<0.001
Na (mmol/L)	138.6±4.5	131.0±5.5	136.3±3.2	139.4±2.7	141.4±2.6	142.9±3.3	145.6±5.1	153.3±8.7	<0.001
K (mmol/L)	4.3±0.7	4.3±0.9	4.3±0.7	4.3±0.6	4.3±0.6	4.2±0.8	4.1±1.0	3.9±0.8	<0.001
HCO_3_ (mmol/L)	22.5±3.9	24.3±5.2	23.7±3.8	22.8±3.1	21.1±3.0	18.9±3.7	16.0±4.8	15.0±6.4	<0.001
Diff_Na-Cl_ (mmol/L)	37.4±3.6	40.8±4.9	38.9±3.1	37.3±2.6	35.6±2.6	33.6±3.2	31.3±4.9	31.3±7.1	<0.001
Cl: Na ratio	0.73±0.03	0.69±0.03	0.72±0.02	0.73±0.01	0.75±0.01	0.77±0.02	0.79±0.03	0.80±0.04	<0.001
Anion Gap (mmol/L)	14.9±2.6	16.5±4.3	15.1±2.8	14.6±2.1	14.5±2.0	14.7±2.3	15.2±2.7	16.3±3.6	<0.001
Albumin (g/dL), n = 2,710	3.6±0.7	3.5±0.8	3.6±0.6	3.8±0.6	3.7±0.7	3.4±0.7	3.4±0.7	3.2±0.8	<0.001

Continuous data are presented as mean±SD; categorical data are presented as count (%)

#### 3-c. Clinical outcomes

**Fig 4:**
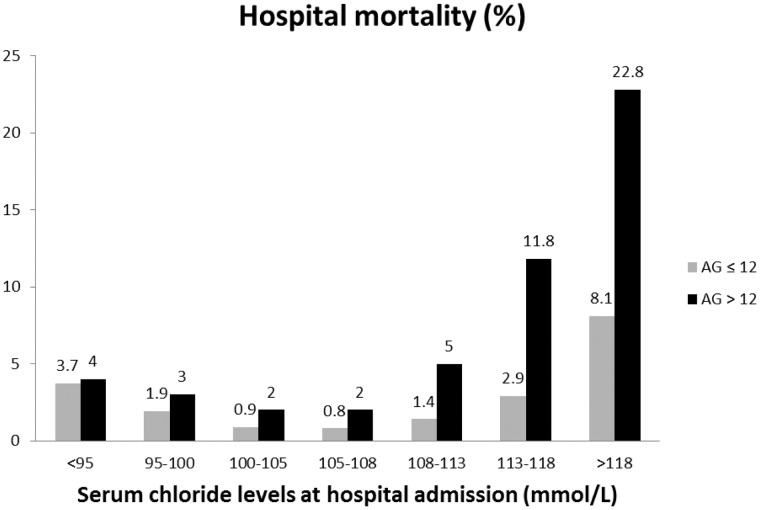
Hospital mortality in percentage (%) among patients with AG ≤12 and >12 mmol/L with various admission Cl levels.

Consistent with previous publications [26–28], patients with elevated AG had a higher overall hospital mortality than those without AG elevation, *p*<0.001. Additionally, we noted a pattern of incremental mortality disparity with increasing sCl (Fig 4, Table 5) that has never been described previously.

**Table 5:** Hospital mortality in patients with AG ≤12 or >12 mmol/L and with various serum Cl concentrations.

Outcome	Admission serum chloride level (mmol/L)						
	<95	95–100	100–105	105–108	108–113	113–118	>118
AG ≤12 (n = 63,630)							
Hospital mortality	80 (3.7)	139 (1.9)	238 (0.9)	122 (0.8)	119 (1.4)	40 (2.9)	18 (8.1)
- Model 1: unadjusted	4.96 (3.71–6.58)	2.50 (1.96–3.20)	1.13 (0.91–1.40)	1 (ref)	1.79 (1.39–2.31)	3.88 (2.67–5.52)	11.47 (6.65–18.70)
- Model 2#	2.87 (2.13–3.85)	1.59 (1.24–2.05)	1.06 (0.85–1.33)	1 (ref)	1.55 (1.20–2.00)	2.96 (2.02–4.24)	6.76 (3.81–11.39)
- Model 3: Model 2 and Na	1.90 (1.30–2.76)	1.32 (1.002–1.73)	0.99 (0.79–1.25)	1 (ref)	1.62 (1.25–2.09)	3.29 (2.24–4.74)	8.28 (4.62–14.09)
AG >12 (n = 13,089)							
Hospital mortality	47 (4.0)	87 (3.0)	117 (2.0)	42 (2.0)	45 (5.0)	13 (11.8)	6 (22.2)
- Model 1: unadjusted	2.01 (1.32–3.08)	1.51 (1.05–2.21)	0.98 (0.69–1.42)	1 (ref)	2.55 (1.66–3.93)	6.51 (3.26–12.21)	13.87 (4.89–34.29)
- Model 2#	1.58 (1.02–2.45)	1.30 (0.89–1.92)	1.01 (0.71–1.46)	1 (ref)	2.11 (1.36–3.27)	4.14 (1.99–8.13)	5.87 (1.91–16.20)
- Model 3: Model 2 and Na	0.92 (0.54–1.56)	0.99 (0.66–1.50)	0.91 (0.63–1.32)	1 (ref)	2.30 (1.48–3.58)	5.45 (2.59–10.84)	11.54 (3.59–33.62)

^#^model 2: Adjusted for age, sex, Charlson comorbidities score, eGFR and principal diagnosis.

In patients with AG ≤12 mmol/L, hospital mortality risk was elevated as the sCl levels deviated from 100–108 mmol/L in all Models (Table 5), consistent with a mortality significance in hypo- and hyperchloremia. In patients with elevated AG, however, sCl <100 and >108 significantly predicted hospital mortality in the unadjusted and adjusted (Models 1 and 2). When further adjusted for dysnatremia, only high sCl (>108 mmol/L) significantly predicted hospital mortality. These results suggest that hyperchloremia (duo acidoses of UMAs and hyperchloremia), but not hypochloremia (mixed UMA acidosis and hypochloremic alkalosis), independently predicted hospital mortality in patients with elevated AG.

### 4. Post-admission Cl evolution and mortality significance

Cl evolution within the first 48hr of hospital admission (n = 55,523) showed that 17.2% (n = 9,553) and 17.2% (n = 9,525) had Cl increase, by 2–4 and by >4 mmol/L, respectively, while 12.2% (n = 6,776) and 9.3% (n = 5,138) had sCl decrease by the same degrees (Table 6). Patients in all categories, except for cardiovascular diseases, showed Cl increase. With rising sCl, hemoglobin concentrations fell, suggesting hemodilution.

**Table 6:** Baseline characteristics in patients with Cl evolution within 48 hours of hospital admission.

variables	Change in serum chloride within the first 48 hours of hospital admission (mmol/L)						
	< -4	-4 to -2	-2 to 0	0 to 2	2 to 4	>4	p
N	5,138	6,776	11,751	12,780	9,553	9,525	
Age (year)	60.3±17.1	63.3±16.7	63.8±16.7	63.8±17.1	63.9±17.4	63.0±17.9	<0.001
Male	2,890 (56)	3,873 (57)	6,643 (57)	7,051 (55)	4,917 (51)	4,486 (47)	<0.001
Caucasian	4,739 (92)	6,331 (93)	11,004 (94)	11,940 (93)	8,868 (93)	8,785 (92)	<0.001
Principal Diagnosis Cardiovascular Endocrine/Metabolic Gastrointestinal Renal Disease Hematology/Oncology Infectious Disease Respiratory Injury/poisoning Other	2,096 61 285 102 577 74 137 835 971	2,215 121 464 157 971 118 237 1,069 1,424	3,344 230 947 315 1,700 306 507 1,808 2,594	3,240 291 1,220 380 1,686 417 651 2,064 2,831	2,053 327 1,272 333 1,138 465 582 1,399 1,984	1,662 618 1,558 548 1,004 857 585 1,265 1,428	<0.001
Charlson Score	1.6±2.2	1.9±2.4	2.0±2.4	2.0±2.4	2.0±2.4	2.2±2.5	<0.001
eGFR (ml/min/1.73m2)	79.0±28.1	75.2±28.1	74.9 (28.1)	74.9±28.7	74.0±29.5	71.1±32.3	<0.001
Na (mmol/L)	139.7±3.6	139.1±3.4	138.6±3.6	137.8±3.9	137.0±4.1	135.2±5.3	<0.001
HCO_3_ (mmol/L)	23.6±3.7	24.6±3.6	25.1±3.5	25.3±3.6	25.4±3.8	25.0±4.4	<0.001
Anion Gap (mmol/L)	7.7±3.8	8.9±3.3	9.3±3.2	9.7±3.3	10.1±3.4	11.2±4.2	<0.001
Change in Hemoglobin (g/dL)	-0.8±1.6	-0.8±1.4	-0.9±1.3	-1.0±1.3	-1.1±1.3	-1.4±1.4	<0.001

Continuous data are presented as mean±SD; categorical data are presented as count (%)

Hospital mortality risk was elevated in patients with sCl increase (>2 mmol/L) in fully adjusted models (Table 7). When patients were grouped based on their admission sCl, sCl increases in all groups (>4 for admission sCl<105, >2 for admission sCl 105–108, and 0–2 for admission sCl >108 mmol/L) independently predicted hospital mortality (Table 8).

**Table 7:** Hospital mortality in patients with Cl evolution within 48 hours of hospital admission.

Outcome	Change in serum chloride within the first 48 hours of hospital admission (mmol/L)					
	< -4	-4 to -2	-2 to 0	0 to 2	2 to 4	>4
Hospital Mortality	68 (1.3)	78 (1.2)	155 (1.3)	178 (1.4)	179 (1.9)	305 (3.2)
Mortality, OR (95%CI)						
- Unadjusted	1.00 (0.75–1.33)	0.87 (0.66–1.14)	1 (ref)	1.06 (0.85–1.31)	1.43 (1.15–1.78)	2.47 (2.04–3.01)
- Adjusted#	1.14 (0.85–1.52)	0.91 (0.68–1.19)	1 (ref)	1.03 (0.83–1.28)	1.33 (1.07–1.66)	2.10 (1.72–2.58)

^#^ adjusted for age, sex, Charlson Comorbidities Score, eGFR, principal diagnosis

**Table 8:** Hospital mortality in subgroups of patients with various serum Cl levels.

Outcome (n = 55,523)	Change in serum chloride within the first 48 hours of hospital admission (mmol/L)					
	< -4	-4 to -2	-2 to 0	0 to 2	2 to 4	>4
Serum Cl <105 mmol/L (n = 33,515)						
Hospital Mortality	13 (1.3)	30 (1.2)	88 (1.5)	120 (1.4)	133 (1.8)	237 (2.8)
Unadjusted OR (95%CI)	0.85 (0.45–1.47)	0.84 (0.54–1.25)	1 (ref)	0.98 (0.75–1.30)	1.25 (0.95–1.64)	1.96 (1.54–2.53)
^#^Adjusted OR (95%CI)	0.87 (0.46–1.52)	0.86 (0.56–1.30)	1 (ref)	1.00 (0.76–1.33)	1.24 (0.94–1.64)	1.84 (1.43–2.39)
Serum Cl 105–108 mmol/L (n = 12,447)						
Hospital Mortality	8 (0.7)	15 (0.7)	29 (0.8)	19 (0.6)	26 (1.6)	37 (4.4)
Unadjusted OR (95%CI)	0.89 (0.38–1.85)	0.83 (0.43–1.53)	1 (ref)	0.74 (0.41–1.32)	2.03 (1.19–3.46)	5.59 (3.42–9.20)
^#^Adjusted OR (95%CI)	0.95 (0.40–1.99)	0.82 (0.43–1.51)	1 (ref)	0.69 (0.38–1.23)	1.80 (1.04–3.10)	4.35 (2.61–7.31)
Serum Cl >108 mmol/L (n = 9,561)						
Hospital Mortality	47 (1.6)	33 (1.6)	38 (1.8)	39 (3.0)	20 (3.1)	31 (9.8)
Unadjusted OR (95%CI)	0.90 (0.58–1.39)	0.89 (0.55–1.42)	1 (ref)	1.72 (1.10–2.72)	1.77 (1.01–3.04)	6.07 (3.70–9.89)
^#^Adjusted OR (95%CI)	1.11 (0.72–1.75)	0.96 (0.59–1.55)	1 (ref)	1.61 (1.01–2.56)	1.51 (0.84–2.63)	4.33 (2.55–7.31)

^#^ Adjusted for age, sex, Charlson comorbidities score, eGFR and principal diagnosis.

**Fig 5:**
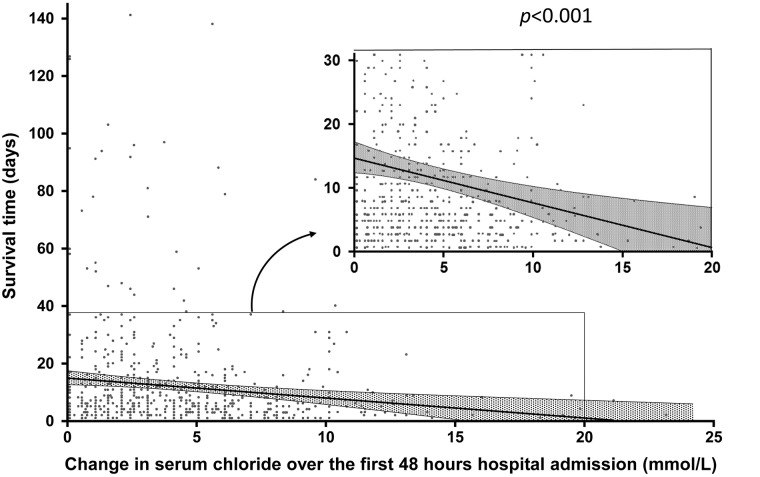
Days of patient survival relating to Cl increase within 48 hours of admission.

To investigate whether the sCl elevation was contributed by Cl-rich fluid administration, we selected 100 patients with minimal sCl change (-2 to 0 mmol/L) and 100 with sCl increase (>4 mmol/L) from the randomized samples (JMP statistical software was used for the randomization) based on the preliminary power calculation. The proportion of 0.9% saline infusion (volumes of infused saline/the total fluid infusion) in the two groups was 37.9 and 59.1%, respectively, p<0.0001, consistent with a larger proportion Cl-rich fluid administration in patients with sCl elevation. An inverse relationship between the magnitude of post-admission sCl increase and the days of patient survival was also found (Fig 5). Our observations were similar to a report showing sCl increase, induced by 0.9% saline, to be inversely related to the survival time in septic rodents [29].

## Discussion

In this large, single center study of all hospital admission, sCl alterations are not only common, but also independently associated with elevated risks for hospital mortality, LOS and discharge to a care facility. Furthermore, in adjusted analysis, post-admission sCl increase, associated with a higher percentage saline infusion, independently predicted fewer days of patient survival, when compared to patients without sCl increase.

sCl is responsible for about one third of the extracellular fluid tonicity and two-thirds of all anionic charges in plasma. Because of its high concentration, sCl is the most important anion to balance the extracellular cations. An increase in sCl out of proportion to Na causes SID reduction and hyperchloremic acidosis. In critically ill patients, saline-driven hyperchloremic acidosis is becoming increasingly recognized and is caused by non-physiological Cl (154 mmol/L) in 0.9% saline. Hypochloremia disproportionate to Na in the absence of UMA, conversely, engenders alkalosis. Acidosis caused by accumulation of UMAs can be detected using SIG[3], which can be accurately reflected by AG corrected for serum albumin [30].

In this study, the distributions of admission sCl are associated with the disease categories with a known propensity for acid-base alterations. For example, most cardiovascular admissions had hypochloremia which is consistent with frequent loop and thiazide diuretics use. Loop and thiazide diuretics disproportionately excrete Cl in relation to Na (in 2:1 and 1:1 ratio, respectively), leading to hypochloremic alkalosis. Patients with gastrointestinal diseases were predominantly hypochloremic, which could have been related to gastric alkalosis in patients with upper gastrointestinal and biliary diseases. Admissions due to respiratory diseases were predominantly hypochloremic, potentially related to a degree of compensatory metabolic alkalosis in the setting of ventilatory insufficiency. Admissions under the category of endocrine/metabolic diseases frequently had hypochloremia, which could have represented patients with uncontrolled diabetes and ketoacidosis (hypochloremia in the setting of ketoacidosis).

Alkalosis is known to increase the occurrence of arrhythmia and mortality in critically ill patients [31]. Clinical effects of acidosis, however, have not been consistent across studies. Transient acidosis in healthy adults is well tolerated. Effects of acidosis in ill adults are controversial. Acidemia can shift the O_2_ dissociation curve to enhance tissue O_2_ delivery. There is, however, strong evidence of a poor prognosis in critically ill patients with lactic acidosis, while hyperchloremic acidosis might not exert a mortality significance [32]. Our results show that sCl alterations (outside the range of 100–108 mmol/L) in patients without AG elevation independently predicted poor outcomes, including hospital mortality. The farther away from 100–108 mmol/L, the worse the prognosis. For those with elevated AG, however, only hyperchloremia, >108 mmol/L, was independently associated with higher mortality. Intriguingly, the difference in mortality between the two AG groups grew progressively with rising Cl (Fig 4). It is tempting to speculate that in patients with hypochloremia and without AG elevation, metabolic alkalosis was dominant, accounting for the mortality risk [31]. In patients with hypochloremia and AG elevation, the dual pathology of hypochloremic alkalosis and AG acidosis potentially offsets the net pH change and thus might have attenuated the mortality consequences. By the same token, in patients with simultaneous AG and serum Cl elevations, the presence of dual AG and hyperchloremic acidoses likely escalated the acidosis severity and worsened mortality. Although lactic acidosis in critical illness is often assumed to reflect tissue hypoperfusion and has been used to direct fluid resuscitation strategies, our study suggests that sCl concentrations are an additional independent predictor for poor outcome.

The post-admission sCl evolution was informative. Except for the cardiovascular category, the majority of patients showed sCl increase. Importantly, regardless of admission sCl values, sCl increase was across the board linked to elevated hospital mortality (Tables 7 and 8). Given the evidence of hemodilution (hemoglobin reduction), the disproportionate sCl increases are consistent with Cl-rich fluid administration. This possibility was confirmed in random samples of patients with and without sCl increase. Notably, the only patient category that did not show sCl increase was cardiovascular, which is consistent with clinical practice as fluid infusion for these patients is typically avoided. Cl increase in septic rodents shortens their survival time [29]. Such inverse relationship was also demonstrated in our cohort (Fig 5). These results suggest that post-admission sCl increase associated with 0.9% saline infusion is detrimental, consistent with the known detrimental effects of hyperchloremia on multiple organ systems [33–40].

There are several limitations in this study. First, although the sample size is large, this is a retrospective cohort study. The data, however, are recent and thus reflect current practice. The short 3-year study duration avoided major change in practice style. Second, the study lacks granularity to examine clinical manifestations of sCl alterations. Clinical manifestations, however, were not a study objective. Third, the cut-off for AG was 12 mmol/L, which was arbitrary. Given that only 17.1% of the total cohort had higher AG, we decided to use a relatively high cut-off to ensure specificity in determining the presence of UMAs. Fourth, the nature of the UMAs was not investigated. Published studies suggest that UMAs are mostly lactic acids and ketoacids, and despite an exhaustive search, not all identities of UMAs can be determined [41]. Fifth, we used surrogates for SID and SIG. Although both surrogates have been extensively validated [5], there could still be a degree of decreased precision. The use of surrogates, however, allowed us to analyze the acid-base status in the vast majority of admissions, minimizing patient selection bias. Sixth, we did not consider the other two independent components (A_TOT_ and pCO_2_) in the Steward model. Among these components, however, SID is by far the dominant and independent determinant. It is not influence by changes in pCO_2_ and A_tot_ [3]. Moreover, most published studies evaluating acid-base balance use SID almost exclusively, making our results comparable with existing publications. Taken together, the robust results from this large contemporary patient sample show unequivocally that Cl alterations impart major outcome significance.

## Conclusions

sCl alterations outside of 100–108 mmol/L range are common at hospital admission and can independently predict poor clinical outcomes, including hospital mortality. Post-admission sCl increase, associated with Cl-rich 0.9% saline infusion, is not only associated with higher hospital mortality, but is also inversely correlated with days of patient survival. Given that sCl values are routinely obtained and available for vast majority of patients, attention should be paid to the sCl value. Although our study results do not establish causality, they do raise an important question of whether Cl-rich fluids compromise patient outcomes. This question should be addressed with future prospective randomized trials.

## Abbreviations

AG: anion gap
A_TOT_: total weak non-volatile acids
CI: confidence interval
IQR: interquartile range
LOS: length of hospital stay
OR: odds ratio
sCl: serum chloride
SID: strong ion difference
SIG: strong ion gap
UMA: unmeasured anion
